# Characterization of Phytoplankton Composition in Lake Maggiore: Integrated Chemotaxonomy for Enhanced Cyanobacteria Detection

**DOI:** 10.3390/microorganisms12112211

**Published:** 2024-10-31

**Authors:** Elisabetta Canuti, Martina Austoni

**Affiliations:** 1 European Commission, Joint Research Centre (JRC), 21027 Ispra, VA, Italy; 2 National Research Council of Italy, Water Research Institute, CNR-IRSA, 28922 Verbania Pallanza, VB, Italy

**Keywords:** CHEMTAX, Lake Maggiore, HPLC pigments phytoplankton, cyanobacteria, bloom, chemotaxonomy

## Abstract

Cyanobacterial blooms in lakes have increased in frequency and intensity over the past two decades, negatively affecting ecological and biogeochemical processes. This study focuses on the phytoplankton composition of Lake Maggiore, with a special emphasis on cyanobacteria detection through pigment composition. While microscopy is the standard method for phytoplankton identification, pigment-based methods provide broader spatiotemporal coverage. Between May and September 2023, five measurement campaigns were conducted in Lake Maggiore, collecting bio-geochemical and bio-optical data at 27 stations. The total Chlorophyll-a (TChl *a*) was measured, with concentrations ranging from 1.13 to 6.9 mg/m^3^. Phytoplankton pigment composition was analyzed using High-Performance Liquid Chromatography (HPLC) and the CHEMTAX approach was applied for phytoplankton classification. The results were cross-validated using Principal Component Analysis (PCA), Hierarchical Cluster Analysis (HCA), and microscopic counts. Cyanobacteria were identified based on unique pigment markers, such as carotenoids. The HPLC-derived pigment classification results aligned well with both PCA and HCA and microscopic counts verified the accuracy of the pigment-based chemotaxonomy. The study demonstrates that pigment-based classification methods, when combined with statistical analyses, offer a reliable alternative for identifying cyanobacteria and other phytoplankton groups, with potential applications in support of remote sensing algorithm development.

## 1. Introduction

Harmful algal blooms (HABs), particularly those caused by Cyanobacteria, have been increasing globally in both magnitude and frequency, posing significant risks to aquatic ecosystems and human health [1,2]. Accurately estimating Cyanobacteria presence is critical for mitigating the risks of cyanotoxin exposure and for evaluating both current and historical water quality trends in inland waters [3,4].

In northern Italy, lakes such as Lake Maggiore have experienced a rising frequency of HABs over recent decades [5,6,7] Lake Maggiore, an oligotrophic lake with a long history of monitoring [8,9,10,11], harbors high biodiversity within its phytoplankton community [12]. Recent studies indicate significant seasonal shifts in this community, with diatoms and cyanobacteria groups dominating during the summer months [13,14] In particular, the increasing presence of small cyanobacteria, such as *Aphanothece* and *Aphanocapsa*, during late summer suggests ecological changes potentially driven by climatic factors and increased water column stability. Continuous monitoring of these changes is essential to better understand their ecological implications.

Traditional microscopy, while reliable for species-level identification, is labor-intensive and lacks the spatial and temporal coverage required for comprehensive lake monitoring [15]. In contrast, chemotaxonomy, which classifies phytoplankton based on pigment composition, offers a rapid and cost-effective alternative [16,17,18]. The detection of cyanobacteria through their unique pigment markers—such as phycobiliproteins and carotenoids (e.g., echinenone, zeaxanthin)—is particularly useful for distinguishing them from other phytoplankton groups [19,20]. Whether the accurate quantification of phycobiliproteins pigments remains challenging due to spectral overlap with chlorophyll a (TChl *a*) [21,22], the quantification of carotenoids and chlorophylls in phytoplankton is a more assessed technique [23]. CHEMTAX algorithm to classify phytoplankton groups based on biomarker pigments [24] has already been successfully applied in marine, estuarine, and freshwater systems [20,25,26,27,28,29,30,31]. However, despite its advantages, chemotaxonomy has limitations, including the challenge of accurately describing the phytoplankton community when some pigments, like fucoxanthin, are common across multiple groups. Furthermore, critical challenges include the need to establish diagnostic pigment ratios that are ecosystem-specific and to ensure that samples are grouped based on similar environmental conditions to avoid misrepresentation of phytoplankton diversity [32,33,34] 

This study aims to apply chemotaxonomy to characterize phytoplankton composition and improve cyanobacteria detection in Lake Maggiore. Unlike previous studies on Lake Maggiore [9,10,11,35,36,37] that relied primarily on traditional microscopic methods, this research leverages high-performance liquid chromatography (HPLC) alongside the CHEMTAX algorithm to classify phytoplankton groups based on biomarker pigments. Furthermore, the findings were validated using Principal Component Analysis (PCA), Hierarchical Cluster Analysis (HCA), and microscopic counts, offering a robust and integrated approach for assessing phytoplankton diversity and cyanobacteria prevalence. By enhancing our understanding of the phytoplankton community structure, this research contributes to the development of more effective water quality monitoring tools, particularly in light of increasing HAB occurrences and their associated risks. Furthermore, the study’s findings could aid the validation of satellite-based bio-optical algorithms for remote sensing applications in HAB detection [38,39,40].

## 2. Methods

Lake Maggiore, the second largest lake in Italy, is a subalpine lake situated at the border between Italy and Switzerland. It stretches over 66 km from the northern Swiss Canton of Ticino to the southern Italian region of Piedmont and Lombardy, with a maximum width of 4.5 km. Lake Maggiore’s bathymetry is characterized by its deep glacial origin, with a maximum depth of 383 m. Hydrologically, Lake Maggiore is part of the Ticino River basin, with the Ticino River being its main tributary and outflow. Other significant tributaries include the Toce River in the northern region and several smaller streams along the western and eastern shores. The climate around Lake Maggiore is classified as temperate, with distinct seasonal variations due to the proximity to the Alps. Lake Maggiore experiences stratification due to temperature and density differences between surface and deep waters from late spring to autumn. Because of its great depth and local weather conditions, the lake does not fully mix every year. It is classified as holo-oligomictic, meaning complete mixing only occurs during very cold and windy winters [41]. The average theoretical renewal time for Lake Maggiore water was estimated to be over 4 years [42]. Oxygenation of the deep waters is partially maintained by oxygen from tributaries. During winter overturns, mixing typically reaches depths of 100–150 m [41]. However, climate change is significantly affecting the lake’s thermal and hydrodynamic properties, with major impacts on its oxygen levels [43,44].

### 2.1. Field Sample Collection

**Figure 1:**
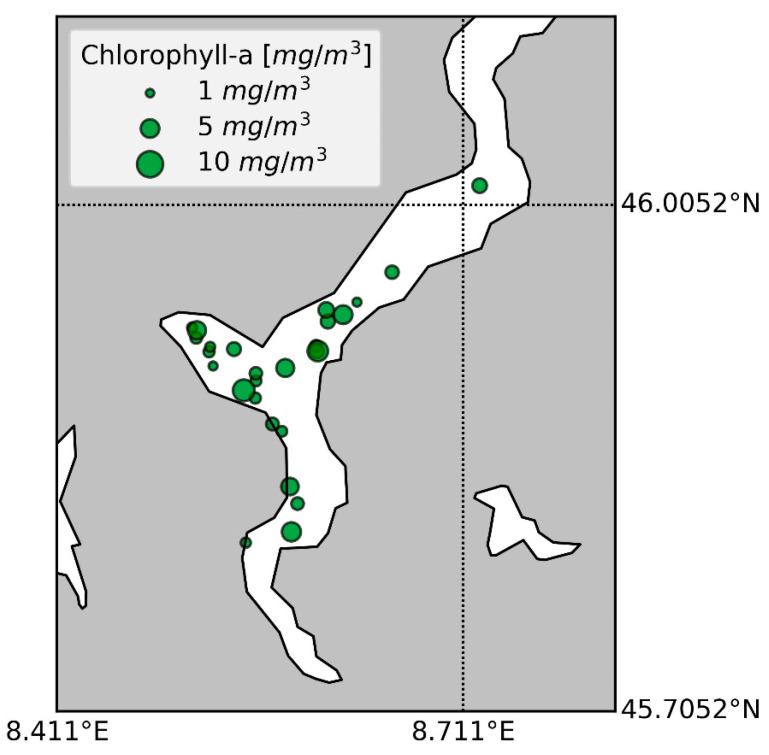
Spatial distribution of the LM23 collecting stations: the size is proportional to the TChl *a* content.

In the period May–September 2023, five measurement campaigns on Lake Maggiore (LM23) were performed for collecting bio-geochemical and bio-optical measurements in support of remote sensing validation activities. The LM23 campaigns consisted of 27 stations (Figure 1), measuring TChl *a*, phytoplankton pigments, and ancillary measurements. In addition, inherent optical properties (particulate absorption coefficient, chromophoric dissolved organic matter) and in-water radiometric measurements (the latter not discussed in the present paper) were collected. The seasons covered in the present study were spring (11 stations, 2 campaigns), summer (11 stations, 2 campaigns), and early autumn (5 stations, 1 campaign). The lake depth in the collection points varied from 60 m to the deepest point of Lake Maggiore (383 m) (Supplementary Figure S1). The LM23 campaigns were designed for satellite validation purposes and sampling points were selected to avoid coastal and bottom influences, ensuring representative mid-lake conditions. Additionally, a station near Ghiffa (LAT 45°.9666, LON 8°.6561), where Ghiffa is considered representative of lake conditions since is the deepest point of the lake (370 m depth), was included in each campaign for consistency across seasons. The time sampling interval was between 9:30 and 15:00 local time, as close as possible to the satellite overpass. The location of the sampling points was selected each time depending on the weather conditions, favoring clear sky and wind-calm conditions, enhancing the possibility of satellite-in situ matchup creation. For each measurement point, 20 L of water was collected 50 cm below the lake’s surface in a pre-rinsed polypropylene container. Samples for SPM, TChl *a*, and algal pigments HPLC analysis were preconditioned onboard and stored in a freezer at −80 °C or liquid nitrogen upon the analysis. Ancillary measurements included surface temperature, suspended particulate matter (SPM), and Secchi Disk readings. Matching samples for microscopic determination were collected, preserved in acetic Lugol’s solution, and stored in the dark at 4 °C until analysis.

### 2.2. HPLC Pigment Dataset

The JRC method is a modification of [45] and is described in detail in [46]. The analysis was conducted using an HPLC/DAD system (HPLC 1200 Agilent Tech., USA), an RP-C8 monomeric column (Zorbax Eclipse XDB-C8, 3.5 μm particle size, 150 × 4.6φ), and an injection of 150 µL sample mixed in a loop with 365 µL of buffer (tetrabutylammonium acetate, 28 mM). The extraction of the natural samples for the JRC was conducted in 2.5 mL of 0.025 g/L of internal standard (Vitamin E acetate, Fluka v.2024.1.2, USA) dissolved in Acetone (HPLC gradient, Merck, Germany) and 150 μL of MilliQ water, soaked for 1 h at −20 °C, sonicated for 90 s in ice with a sonication probe (Bandelin, Germany), soaked for 3.5–4 h at −20 °C, and then clarified by extraction through a 0.2 μm Teflon Syringe prior to injection.

To calibrate the HPLC, pigment standards from DHI Lab Products (Denmark) were used. The calibration curve consisted of nine points, covering a range of concentrations from a dilution close to three times the Signal-to-Noise Ratio (SNR) concentration to the standard concentration, as described by [47]. The linearity of the calibration curves was verified across the entire analysis range. The Low Limit of Detection (LOD) was defined as three times the instrumental SNR at each quantification wavelength. Peaks below the LOD are considered unidentified. The internal standard was detected at 222 nm, while chlorophyll *a* and chlorophyll *a* epymers and allomers were detected at 665 nm. All other pigments were detected at 450 nm.

The pigments that could be identified and quantified by the analysis of the natural samples were 19’-hexanoyloxyfucoxanthin (Hex), 19’-butanoyloxyfucoxanthin (But), alloxanthin (Allo), fucoxanthin (Fuco), peridinin (Perid), diatoxanthin (Diato), diadinoxanthin (Diadino), Zea, divinyl chlorophyll *a* (DVchl*a*), monovinyl chlorophyll *a* (MVchl*a*), total chlorophyll *b* (TChl *b*), chlorophyll c1 + c2 (Chlc1c2), chlorophyll c3 (Chlc3), neoxanthin (Neo), violaxanthin (Viola), prasinoxanthin (Pras), Lutein (Lut), α,β-carotene (α-caro), β,β-carotene (β-caro), pheophorbide *a* (Pheo), pheophythin *a* (Phy). chlorophyllide *a* (Chlide *a*), Echin, Myxo, Anthe, and Gyro. Following the commonly adopted nomenclature, TChl *a* is intended as the sum of MVchl*a*, DVchl*a,* and Chlide a, and Caro is the sum of α-caro and β-caro [23].

In the chemotaxonomic interpretation via biomarker pigments applied to marine water [16,17,18,48] Diatoms are represented primarily by pigments such as Fuco, Diad, Diat, and various chlorophyll *c* derivatives. Dinophyceae are most closely associated with Peri and Diad. The phylum of Haptophyta is represented by Hex and TChl c1-c2. Chlorophyceae and Prasinophyceae are indicated by pigments such as Pras, Lut, and TChl *b*. Euglenophyceae, on the other hand, are associated with pigments like TChl b. Cyanobacteria are primarily linked to Zea and DVChl *a* and DVChl *b*, while Cryptophyceae are represented by Allo and TChl *c*. Chlide *a* is identified as a marker for the senescence of diatoms, indicating the aging and breakdown of these algae [23]. Zea and Echin are found to be representative of the pico-fraction and Cyanobacteria as secondary pigments, respectively [49]. In lakes [20], Myxo, Zea, Gyro, and Anthe were also associated with the Cyanobacteria population. Echin was found in *Anabaena* and *Dolichospermum* genera and *Anabaena variabilis* Kützing ex Bornet and Flahault [50]. *Dolichospermum lemmermannii* (Richter), pertaining to P.Wacklin, L.Hoffmann, and J.Komárek, has also been recognized in Lake [4,5,8,9,10,11,14,51]. In addition to *Dolichospermum lemmermannii*, the chroccocales cyanobacteria *Microcystis aeruginosa* (Kützing) Kützing, which has a high content of Echin and Myxo, was also identified in Lake Maggiore.

Pigment analysis also differentiated between photoprotective carotenoids (PPC) and photosynthetic carotenoids (PSCs). PPC, comprising Allo, Diad, Diato, Zea, and Caro, plays a crucial role in protecting phytoplankton from excessive light and oxidative stress. On the other hand, PSCs, including But, Fuco, Hex, and Peri, are primarily involved in the light-harvesting process for photosynthesis. In Lake Maggiore samples, But was not present and Hex could be considered as not significant due to its low concentration. The total accessory pigments (TAcc) were calculated as the sum of PPC, PSC, TChl b, and TChl c.

The phytoplankton proportion factor was introduced by Hooker et al., 2005, as a proxy of the different phytoplankton cell size fractions. The Microplankton Proportion Factor (mPF) is determined by the combined concentrations of Fuco and Peri, both of which are pigments commonly associated with larger phytoplankton, such as diatoms and dinoflagellates. This index provides an estimate of the relative abundance of microplankton, organisms typically larger than 20 μm. A higher mPF value indicates a greater presence of these larger phytoplankton, suggesting conditions favorable for species that thrive in environments with ample nutrients and light availability. The Nanoplankton Proportion Factor (nPF) focuses on pigments associated with smaller phytoplankton, specifically Hex, But, and Allo. Nanoplankton, ranging from 2 to 20 μm, often include species belonging to the phyla of Haptophyta and the class of Cryptophyceae, which, in the case of Lake Maggiore, are indicative of oligotrophic conditions especially during periods of low water mixing [7]. The nPF value reflects the relative contribution of these intermediate-sized phytoplankton to the total phytoplankton community. Lastly, the Picoplankton Proportion Factor (pPF) is derived from the concentrations of Zea and TChl *b*. Zea is a key pigment in Cyanobacteria and Prochlorophytes, while chlorophyll *b* is found in green algae. These pigments are indicative of picoplankton, the smallest phytoplankton group, usually less than 2 μm in size. A higher pPF value suggests a predominance of these tiny, often photosynthetically efficient, organisms, which are well-suited to low-nutrient environments or deeper water layers where light is limited.

The HPLC dataset included the pigment characterization of the twenty-seven LM23 stations and of pure cultures of *Anabaena* sp. PCC 7120 and *Microcystis aeruginosa.*

### 2.3. Methods in Data Analysis

#### 2.3.1. HPLC Dataset Quality

The criteria established by Aiken et al. [52] and used for assessing the quality of datasets for bio-optical algorithm development [18] (were applied to evaluate the internal consistency of the LM23 database. The co-variation in log-transformed total chlorophyll a (TChl *a*) and the sum of accessory pigments (TAcc) [53] was verified. The other criteria included i. the correlation between TChl *a* and TAcc, with a variance (r^2^ > 0.9) and a slope within the range of 0.7–1.4; ii. the difference between TChl *a* and TAcc being less than 0.3 TPig; and iii. 85% of the collected stations that satisfy the first two criteria.

#### 2.3.2. Biomass of Individual Plankton Groups

CHEMTAX, developed by [24], is a powerful tool for estimating the biomass of the individual phytoplankton groups as TChl *a*. The composition of phytoplankton communities is based on pigment concentrations measured by HPLC. The CHEMTAX algorithm utilizes an iterative process to refine the initial ratio matrix (F0), adjusting it based on the actual pigment concentrations observed in the samples. Through this iterative adjustment, CHEMTAX minimizes the discrepancy between measured and estimated pigment concentrations, ultimately converging on a solution that best represents the phytoplankton community structure. Our analysis included 12 key pigments: TChl *a*, TChl *b*, TChl *c*, Fuco, Zea, Peri, Allo, Diad, Echin, β,β-Caro, Chlide *a*, and Chl *c3*. These pigments were selected based on their specific association with different phytoplankton groups and their relevance in the ecological study of Lake Maggiore. The 6 phytoplankton groups considered in our analysis were *Bacillariophyceae* (Diatoms), *Cyanobacteria*, *Chlorophyceae*, *Cryptophyceae*, *Dinophyceae*, and *Chrysophyceae*. These groups included a broad range of the primary producers present in Lake Maggiore, providing a comprehensive overview of the phytoplankton community structure. The F0 matrix (Supplementary Table S1) was built using the available lake literature [20]. To account for the inherent variability and potential inaccuracies in the pigment ratio matrix, we set the ratio limit matrix to the default value of 500%. This conservative approach allowed for a broad range of potential ratios, thereby accommodating natural variability and reducing the risk of introducing bias from overly restrictive limits.

#### 2.3.3. Hierarchical Cluster Analysis (HCA)

A hierarchical cluster analysis was performed on the HPLC pigment dataset, with pigment values normalized to TChl *a* (e.g., Fuco a ratios). This method, utilizing Ward’s linkage approach (minimizing inner squared distances), was based on correlation (R, Pearson correlation between phytoplankton pigment ratios) distance calculations. This approach aligns with methodologies used by Latasa et al. [54] and Catlett et al. [55]. A dendrogram was constructed with a cutoff distance of 0.5 to segment the dataset into distinct phytoplankton community clusters. Each sample was subsequently assigned to one of these clusters based on the calculated correlation distances

#### 2.3.4. Principal Component Analysis (PCA)

The Principal Component Analysis (PCA) was applied to the HPLC pigment dataset to decompose the data into orthogonal modes, which represent distinct patterns within the dataset. This method leverages eigenvalues to quantify the variance captured by each mode, where the majority of the dataset’s variability is often explained by the first few components. Previous studies [26,27,28,29,30,31,32,33,34,35,36,37,38,39,40,41,42,43,44,45,46,47,48,49,50,51,52,53,54,55,56,57,58,59] have successfully employed PCA to reveal spatial and temporal patterns in pigment data. For this analysis, the dataset comprised 16 variables (pigments normalized to TChl *a*), and the decomposition was conducted using a Singular Value Decomposition (SVD), as follows:

(1) $$X=U\Sigma V^{T},\;\mathrm{where}x_{ij}\;=\;\Sigma_{k=l,N}u_{ik}\sigma_{k}v_{kj}$$

The matrix U contains the principal components (loadings), and the eigenvalues in Σ represent the variance explained by each mode. The PCA modes allow for a clear visualization of the dominant structures in the data, but it is important to note that this approach does not make assumptions about the covariance between pigments. Though already used, further comparisons with alternative methods are needed to assess its full potential of application of PCA analysis in this context.

#### 2.3.5. Network Community Detection Analysis (NCA)

Network-based community detection was performed on the phytoplankton pigment data using techniques from Kramer et al. [58] and Canuti et al. [59]. In this approach, pigments were represented as nodes in a network, with edges depicting the co-occurrence or similarity between pigments across different stations. Edge weights, calculated using Pearson’s correlation coefficients of normalized pigment ratios, were used to create a similarity matrix, as follows:

(2) $$s_{ij}=|corr(x_{i},x_{j})|$$

This similarity matrix was transformed into an adjacency matrix, which formed the basis of the Weighted Gene Network Community Analysis (WGNCA) [60]. WGNCA uses a beta parameter (set to 6 in this study) to control the influence of edge weights, enhancing the detection of meaningful pigment communities, as follows:

(3) $$a_{ij}={(s_{ij})\;}^{\beta }$$

The Louvain method [61] was employed to maximize network modularity, grouping pigments into distinct communities based on their connectivity. A modularity score greater than 0.3 indicated strong clustering, with well-defined internal community structures and weak external connections.

#### 2.3.6. Microscopy Phytoplankton Determination

In this study, phytoplankton samples were taken at 50 cm below the lake surface. Phytoplankton determinations were carried out on subsamples preserved in acetic Lugol’s solution by placing a defined volume (between 5 mL and 25 mL) into a sedimentation chamber, following the Utermöhl technique [62,63], according to CEN 15204 [64] and classifying the taxa to the species level, where possible. Phytoplankton biovolume was calculated by approximating the shape of the algae to rotational solids; therefore, relative phytoplankton biomass was estimated from the density data with the original measurements of the species biovolume, where each taxon is associated to a geometric shape following Hillebrand et al. [65] and Sun and Liu [66] according to CEN 16695 [67].

## 3. Results

### Dataset Overview

In our analysis, we examined pure species of *Anabaena* sp. PCC 7120 and *Microcystis aeruginosa* (Kützing), reporting their pigment concentrations in [ng/inj] and ratios to TChl *a* and β-caro (Table 1). For *Microcystis aeruginosa*, Myxo was found at 6.3 ng/inj, accounting for 3.0% of TChl a and 37.3% of β-caro. Zea was present at 27.1 ng/inj, representing 12.9% of TChl *a* and 160.4% of β-caro. Gyro was detected at 2.1 ng/inj, making up 1.0% of TChl *a* and 12.4% of β-caro. Echin showed a concentration of 5.2 ng/inj, constituting 2.5% of TChl *a* and 30.8% of β-caro. Lastly, β-caro itself was measured at 16.9 ng/inj, equating to 8.1% of TChl *a*. The relatively high concentration of Zea suggests its critical role in the light-harvesting process and protection against oxidative stress. The high concentration of Zea in particular highlights its significant presence and role within *Microcystis aeruginosa*, while the levels of other pigments like Myxo, Gyro, and Echin provided a detailed understanding of the pigment profile associated with this cyanobacterial species. From the microscopy analysis, *Microcystis* *aeruginosa* were present in the samples collected in July–August 2023. In contrast, *Anabaena* sp. PCC 7120 showed a much higher concentration of β-caro at 29 ng/inj, which constitutes 15.2% of TChl *a*. This higher proportion indicated a greater reliance on β-caro, potentially for photoprotection or structural purposes. Interestingly, while *Anabaena* sp. PCC 7120 exhibits a lower amount of Zea (2.1 ng/inj), it also had a markedly higher concentration of Echin at 22.4 ng/injection, making up 11.7% of TChl *a* and 77.2% relative to β-caro. This suggested that Echin plays a more significant role in *Anabaena* sp. PCC 7120 compared to *Microcystis aeruginosa.*

**Figure 2:**
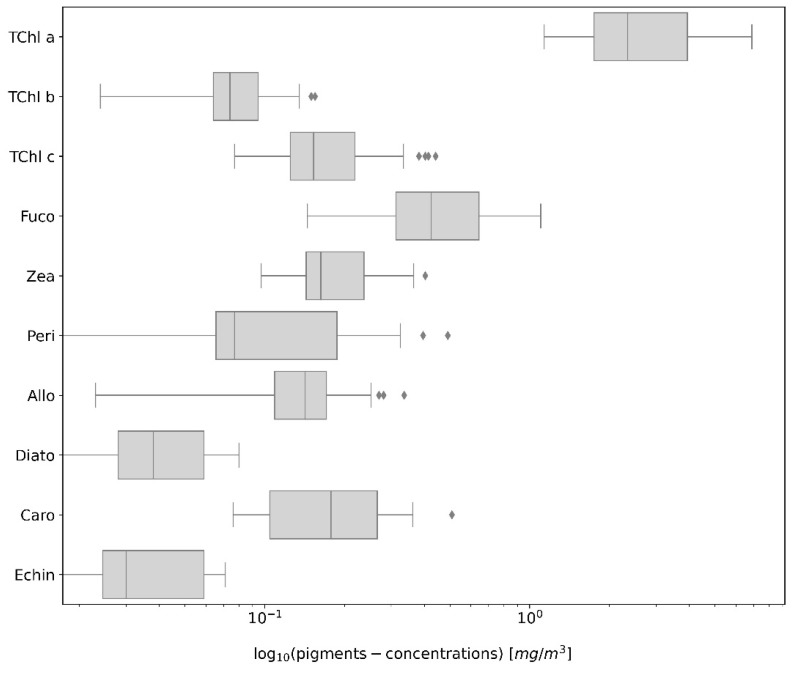
Boxplot of the pigment concentrations log_10_-transformed) of Lake Maggiore 2023 (LM23) campaigns. Each box shows the interquartile range (IQR), with the central line indicating the median concentration. Whiskers extend to 1.5 times the IQR, with outliers represented by dots beyond this range.

The variability of pigments in Lake Maggiore was analyzed by considering various statistical metrics including average concentrations, coefficients of variation (CV%), maximum and minimum values, and the number of measurements lying under the detection limit (Table 2). The results showed significant variability across different pigments (Figure 2), which provided insights into the phytoplankton dynamics and ecological conditions of the lake. Moreover, of the biomarker pigments considered, the Fuco, Allo, and TChl *b* were isolated in almost all the samples, Peri in 76% and Zea in 68% of the samples, while Hex and But were identified in 11% and 5% of the samples, respectively. Both Zea and Echin are markers for cyanobacteria, providing information on their abundance and distribution. The documented pathway involving β-caro as a precursor for Zea [47] highlights the importance of carotenoids in cyanobacterial physiology. The detection of Echin was particularly noteworthy due to its association with cyanobacteria *Dolicospermum* and *Anabaena* species, a cyanobacterial population present in Lake Maggiore. In contrast, the analysis did not detect Myxo and Anthe in natural samples, pigments also linked to cyanobacteria. Their absence may point to either a low abundance of these pigments or particular cyanobacterial species present in the Lake Maggiore.

TChl *a* exhibited an average concentration of 2.933 mg/m^3^, with a CV% of 54.79%. This moderate variability indicated relatively consistent primary productivity levels across different sampling sites. The concentrations ranged from 1.133 mg/m^3^ to a maximum of 6.901 mg/m^3^. The TChl *a* concentration was higher in the samples collected in the afternoon compared to the station in the morning (Supplementary Figure S2). TChl *b* had a lower average concentration of 0.0789 mg/m^3^ and a CV% of 42.6%, indicating less variability compared to TChl *a*. The values ranged from 0.024 mg/m^3^ to 0.155 mg/m^3^ and were present in all of the samples analyzed, which points to a consistent but lower abundance of green algae in the lake. TChl *c* showed an average concentration of 0.1976 mg/m^3^ and a CV% of 55.2%, with values ranging between 0.077 mg/m^3^ and 0.442 mg/m^3^. Fuco, indicative of diatom species, had an average concentration of 0.5131 mg/m^3^ and a CV% of 55.9%. Its concentration varied from 0.145 mg/m^3^ to 1.101 mg/m^3^, reflecting significant spatial variability. No measurements lower than the detection limit were recorded, highlighting the consistent presence of Diatoms. Zea, associated with Cyanobacteria, had an average of 0.1991 mg/m^3^ and a CV% of 42.6%. The concentrations ranged from 0.097 mg/m^3^ to 0.404 mg/m^3^, indicating moderate variability and ubiquitous detection in the samples, pointing to a consistent presence of prokaryotic phytoplankton. Peri, linked to Dinophyceae, exhibited substantial variability with an average concentration of 0.1365 mg/m^3^ and a high CV% of 95.2%. Its values ranged to 0.491 mg/m^3^, with one measurement lower than the limit of detection, indicating patchy distribution and episodic blooms. Allo, indicative of Cryptophyceae, had an average of 0.1467 mg/m^3^ and a CV% of 52.3%, with concentrations between 0.023 mg/m^3^ and 0.336 mg/m^3^. The pigment had a widespread presence across the lake. Diato showed an average of 0.0405 mg/m^3^ and a CV% of 58.1%. The max concentration was at 0.08 mg/m^3^, with four samples where Diato was not identified, indicating a more sporadic distribution of certain phytoplankton groups. Caro had an average concentration of 0.1956 mg/m^3^ and a CV% of 55.9%, with values between 0.076 mg/m^3^ and 0.509 mg/m^3^. Echin had an average of 0.0381 mg/m^3^ and a CV% of 54.2%. The concentrations ranged to 0.071 mg/m^3^ and were not quantified in two stations, indicating some spatial heterogeneity. SPM had the lowest average concentration of 0.0119 g/m^3^ and the lowest CV% of 38.93%, with values ranging from 0.007 g/m^3^ to 0.025 g/m^3^, indicating relatively stable particulate levels in the lake. Geographical coordinates (LAT and LON) for the sampling stations were also recorded, showing a range of latitudes from 45.482 to 45.559 and longitudes from 8.305 to 8.347, covering the spatial extent of the Italian side of Lake Maggiore. The water temperature at the surface varied from a maximum of 25.6 °C to a minimum in April of 15.5 °C. The sampling station corresponds to the shore (60 m) to the deepest bottom (383 m) of Lake Maggiore.

**Figure 3:**
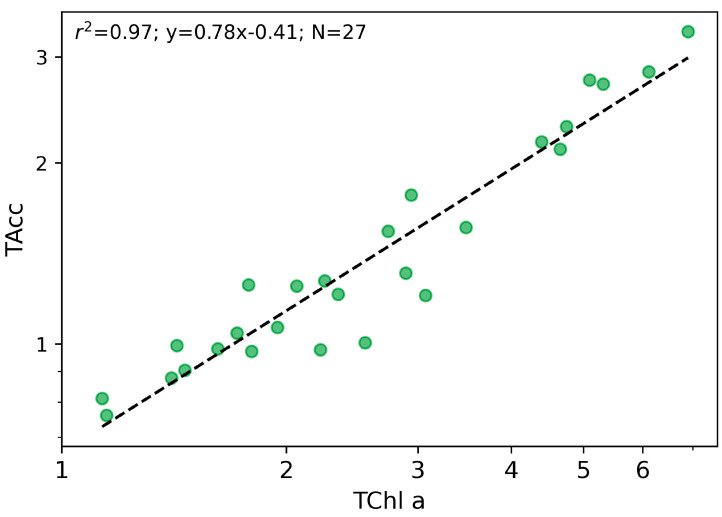
The log–log correlation TAcc/TChl *a* along the five Lake Maggiore Campaigns; the axis in log scale (mg/m^3^). The green dots are the 27 stations of the five campaigns on Lake Maggiore.

The HPLC dataset was tested for quality assurance following the Aiken criteria. A strong log-correlation relationship was observed between TChl *a* and TAcc (Figure 3), with a correlation coefficient (r^2^) of 0.88. The slope of this relationship (0.76) fell within the Aiken criteria (range 0.7–1.4), and the mean differences between TChl *a* and TAcc were lower than 30%, which indicates the consistency and reliability of the measurements and their interpretations (Figure 3).

The analysis of variance (ANOVA) and Tukey tests (Table 3) were conducted on LM23 samples to analyze seasonal variations in measured parameters. Among the pigments, TChl *a* exhibited a notable seasonal effect (*p* = 0.006), with higher concentrations observed in spring compared to fall (Tukey, *p* = 0.0045), although no significant differences were detected between summer and the other seasons. TChl *b* showed no significant seasonal variation (*p* = 0.420). For the total TChl *c*, significant seasonal differences were observed (*p* = 0.0003), with meaningful pairwise differences across all seasons (spring vs. fall, summer vs. fall, and spring vs. summer). Fuco exhibited moderate seasonal variation (*p* = 0.045), with significantly higher values in spring compared to fall (Tukey, *p* = 0.036). Zea showed a strong seasonal effect (*p* = 0.0017), with concentrations in spring significantly higher than those in fall (Tukey, *p* = 0.0015). Similarly, Peri displayed highly significant seasonal differences (*p* = 0.0001), with fall consistently exhibiting lower concentrations than both spring and summer. Allo also demonstrated strong seasonal variation (*p* = 0.002), with significantly higher concentrations in spring compared to both fall and summer (*p* = 0.0041). Caro followed a similar pattern, with strong seasonal effects (*p* = 0.0017) and lower concentrations in fall relative to spring (*p* = 0.0014). Echin showed significant seasonal differences, specifically between fall and spring (*p* = 0.025). However, parameters such as diatom-specific pigments (Diato) and SPM showed no significant seasonal variation.

**Figure 4:**
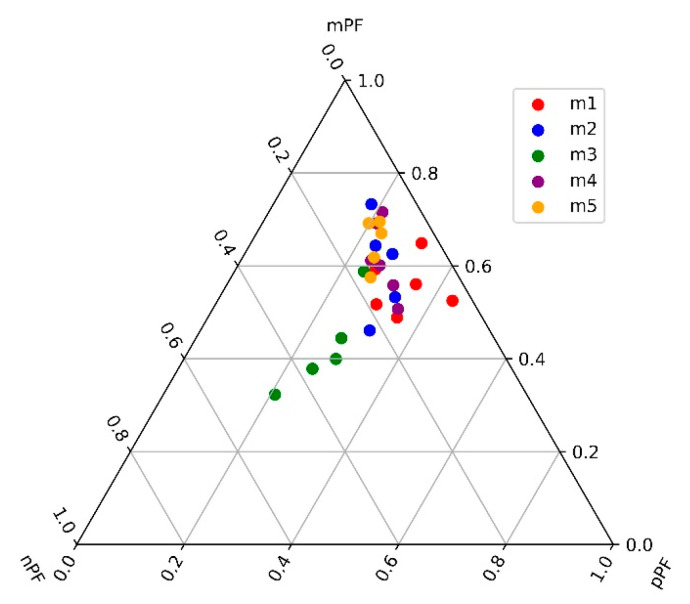
Ternary plot of functional Indices pPF, nPF, and mPF for LM23 campaigns.

From the ternary plot of pPF, nPF, and mPF (Figure 4) it emerged that most of the samples cluster toward the middle-to-upper portion of the triangle, indicating that microplankton (mPF) generally has a significant presence in these samples. The points were concentrated around the 0.4 to 0.8 range on the mPF axis, suggesting that microplankton often constitute a substantial proportion of the phytoplankton community in Lake Maggiore. However, there is also a notable spread toward the lower part of the plot along the nPF and pPF axes, particularly for the m4 group (green dots). This suggests that in some samples, nanoplankton and picoplankton are more dominant, with these points reflecting a more balanced or even pico-/nanoplankton-dominated community. The color-coded points (m1 to m5) indicated different monthly campaigns, showing that there were distinct patterns in the phytoplankton community structure. For example, the m4 group (green, the month of July) tends to have lower mPF values, indicating a lower contribution of microplankton and a higher presence of either nanoplankton or picoplankton. In contrast, the other groups (e.g., m1, m2, m3, and m5) show varying degrees of microplankton dominance. Overall, the plot reflected the diversity in phytoplankton size classes within Lake Maggiore, with certain samples showing a strong microplankton presence while others demonstrate a more balanced or smaller-sized phytoplankton community.

**Figure 5:**
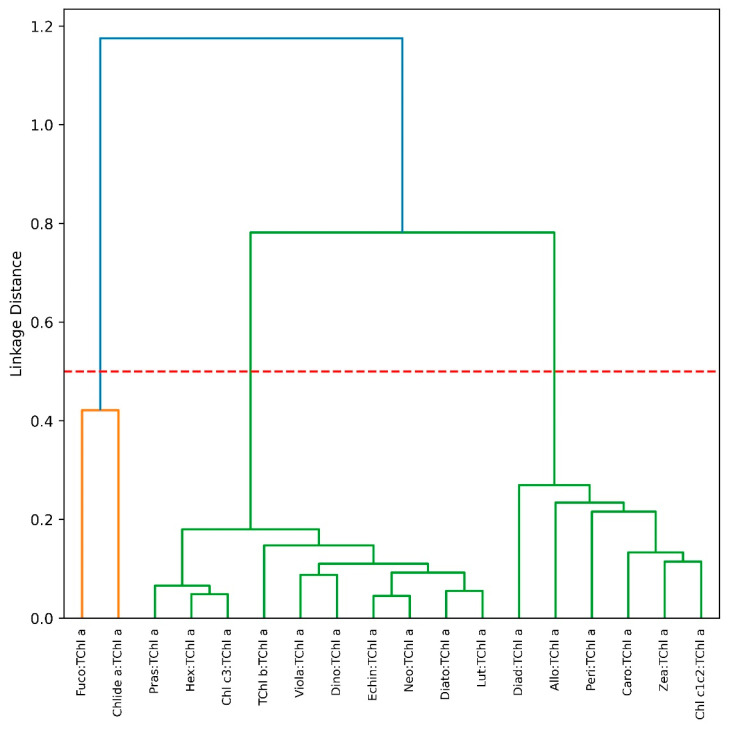
Hierarchical clustering of phytoplankton pigment ratios to TChl *a* for the LM23 dataset. The three-seizes major pigment communities (micro-, nano-, and pico-phytoplankton, from left to right) are identified based on a linkage distance cutoff of 0.5 (red dashed line).

The dendrogram provided a visual representation of the hierarchical clustering of various phytoplankton pigments based on their similarity in taxonomic distribution (Figure 5). The *y*-axis, labeled “Linkage Distance”, indicated the degree of similarity or difference between the pigments, with shorter linkage distances representing closer associations. The dendrogram showed a primary split into three major clusters at a linkage distance of 0.5, as marked by the red dashed line. The first cluster on the left included pigments that were closely associated with micro-phytoplankton and diatom senescence, with Chlide *a* being a prominent representative. This cluster also included Fuco, which, like Chlide *a*, was a pigment strongly linked to diatoms [23]. The second and third major clusters on the right were more diverse, comprising pigments that were distributed across a broader range of phytoplankton groups. Within these clusters, smaller sub-groups can be identified. For example, pigments such as Pras, Viol, Hex, and Neo were linked at a closer distance, indicating that they were predominantly associated with Chlorophyceae. Another distinct grouping included Peri, which was uniquely representative of Dinophyceae and Zea, which is usually associated with prokaryotes such as pico-phytoplankton.

The analysis of PCA (Supplementary Figure S3) showed how different pigments, associated with specific phytoplankton groups, contributed to the overall composition and variance in the dataset. TChl *a*, TChl *b*, Fuco, and Zea were positioned near the origin, suggesting that these pigments were consistently present across all sampling campaigns, with limited variation across different environmental conditions.

**Figure 6:**
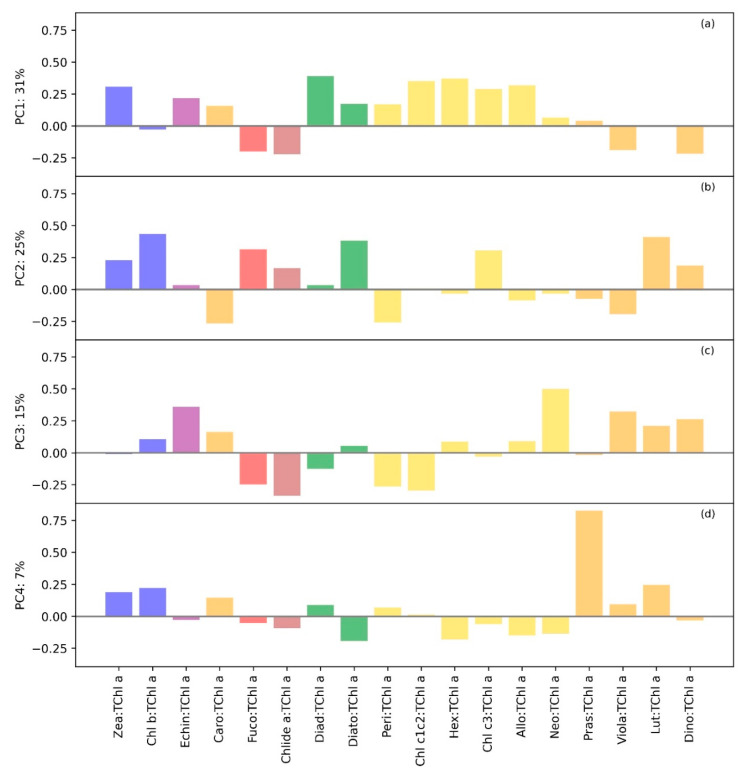
The loadings corresponding to the principal component modes for the pigments ratio to the TChl *a* are shown in panels (**a**–**d**) for the LM23 dataset.

Surface temperature (TSurf) and Allo showed a strong positive correlation along PC1, indicating that higher surface temperatures were associated with increased concentrations of these pigments, particularly during the m5 campaign. In contrast, the depth exhibited a negative correlation with both PC1 and PC2, linking it with lower pigment concentrations, particularly in samples from the m1 campaign, which experienced cooler water conditions. The PCA also applied to the pigment ratio, with the TChl *a*: the contribution of each ratio being considered in terms of loadings (Figure 6). The Principal Component 1 (PC1) was largely influenced by the ratios of Diad:TChl *a*, Hex:TChl *a*, and Chl c1c2:TChl *a*. These pigments were significant markers for various phytoplankton groups, with Diad being indicative of diatoms and Hex-Fuco often associated with certain flagellates. Additionally, the ratios of Zea and Allo further underscored the contribution of Cyanobacteria and Cryptophyceae, respectively. On the other hand, negative loadings for Chlide *a* and Fuco suggested an inverse relationship, where the senescence of diatoms (indicated by Chlide *a*) and the presence of fucoxanthin-rich diatoms were less dominant in explaining PC1. PC2 was primarily driven by the ratio of TChl *b*:TChl *a*, highlighting its role in differentiating green algae, particularly chlorophytes, within the phytoplankton community. Diatom-associated pigments like Diato:TChl *a* and Fuco:TChl *a* also contribute positively to PC2, emphasizing their importance in this secondary source of variance. Conversely, Peri and Caro, the latter being a precursor of Zea and Echin, showed negative loadings. This suggested that while green algae and diatoms were prominent in PC2, dinoflagellates marked by Peri and Caro pigments were inversely related to this component. PC3 highlights the ratio of Neo to TChl *a* as a critical factor, marking the presence of certain chlorophytes. The positive loading of Echin:TChl *a*, a secondary pigment for cyanobacteria, along with Lut:TChl *a*, suggested an important role for these groups in PC3. The negative loadings for Chlide *a* and Peri further delineated the distinct variance pattern, where diatom senescence and dinoflagellate presence are inversely related to the positively loading pigments. PC4 was significantly characterized by the ratio of Pras:TChl *a*, highlighting prasinophytes as a crucial component of the phytoplankton community. The positive contributions of Lut:TChl *a* and TChl *b*:TChl *a* indicated the role of green algae and related groups. Conversely, negative loadings for Hex:TChl *a* and Allo suggested that while Chlorophyceae were prominent, the presence of certain flagellates and Cryptophyceae was less influential in this component.

**Figure 7:**
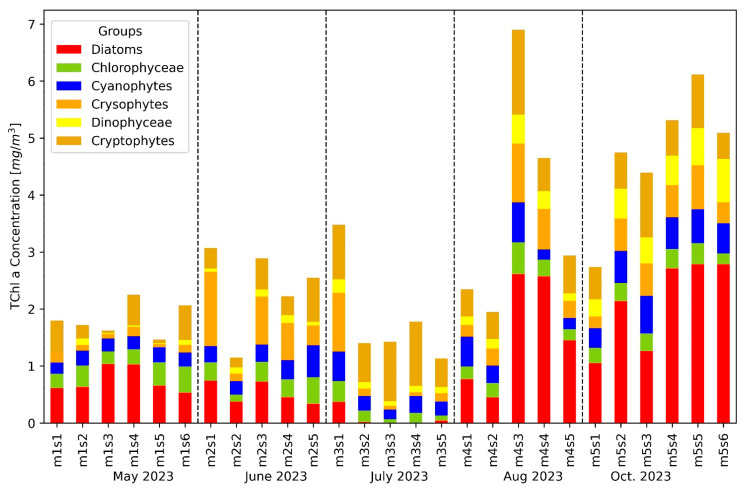
The algal group composition at various stations in Lake Maggiore is determined by CHEMTAX analysis, with each bar representing a specific station. The height of each bar indicates the TChl *a* concentration in mg/m^3^, and the stations are organized chronologically from May to October, as indicated by the vertical dashed lines separating each month.

The findings of unsupervised machine learning (HCA and PCA) were compared with the findings of the CHEMTAX analysis (Figure 7). The final matrix (F1, Table 4) was used for analyzing the whole LM23 pigments dataset. The dominant communities found through CHEMTAX analysis were Diatoms (Bacillariophyceae), Cryptophyceae, and Chrysophyceae, followed by Chlorophyceae and Cyanobacteria. The TChl *a* concentration showed a clear temporal variation across the months (Supplementary Figure S2). Early months (May to June) exhibited lower TChl *a* concentrations compared to later months (July to October), with an increase starting in July. Regarding the algal group CHEMTAX composition, diatoms (represented in red) dominate the phytoplankton community throughout the sampling period, especially in the early months, contributing significantly to the TChl *a* concentration. Chlorophyceae (green) were present in all months but showed only a minor contribution, with their presence remaining relatively stable across different stations. Cyanobacteria (blue) showed a more substantial presence from August to October, indicating a possible seasonal bloom. Dinophyceae (light yellow) contributed minimally across most stations but showed slight increases in the later months. Cryptophyceae (light brown) exhibit significant contributions starting in July and continue to be prominent in the later months, suggesting favorable conditions for their growth during this period.

Upon examining the monthly trends, May (stations m1s1 to m1s6) was characterized by lower TChl *a* concentrations with a strong dominance of diatoms. June (stations m2s1 to m2s5) showed similar patterns with low overall TChl *a* concentrations and diatom dominance. In July (stations m3s1 to m3s5), there was a marked increase in TChl *a* concentrations, with a more diverse algal community, including significant contributions from Dinophyceae and Cryptophyceae, while Diatoms were almost absent. August (stations m4s1 to m4s5) saw further increases in TChl *a* concentrations, with continued high contributions from Diatoms and an increase in Cyanobacteria. October (stations m5s1 to m5s6) maintained high TChl *a* concentrations, with a notable presence of Cyanobacteria and Dinophyceae.

**Figure 8:**
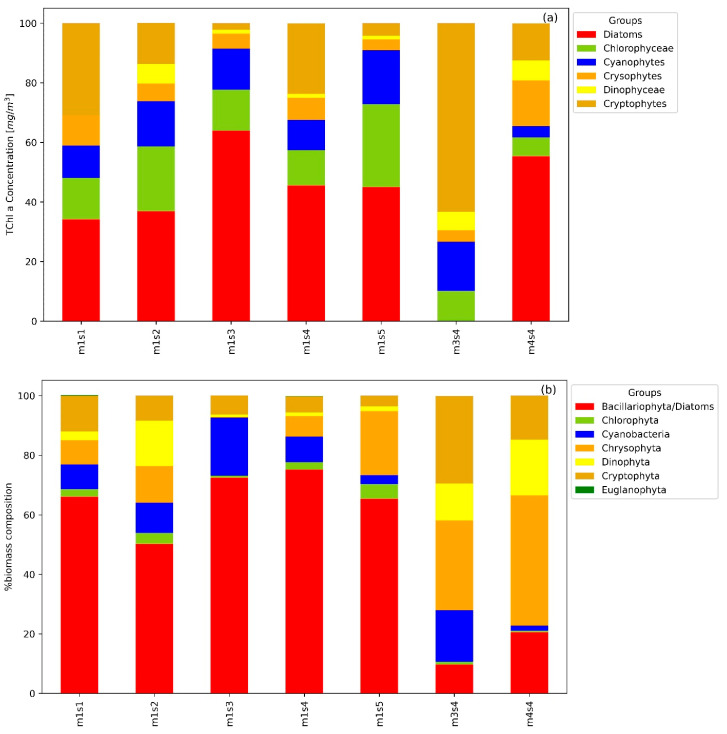
CHEMTAX distribution (**a**) and corresponding microscopy determination (**b**) for matched stations.

The phytoplankton composition in Lake Maggiore was analyzed by microscopic determination (Figure 8b) and was compared with the CHEMTAX analysis for matching stations (Figure 8a). Each bar represents a station, with the algal group composition shown as a percentage of total biomass for the microscopy determination and as TChl *a* concentration for CHEMTAX.

In the microscopy analysis, the dominant groups across most stations were Bacillariophyceae (Diatoms, in red) and Chrysophyta (in brown). Notably, at all the stations of the m1 campaign, Bacillariophyta makes up the majority of the biomass, whereas stations m1s2 showed significant contributions from Cyanobacteria (blue) and m1s5 Chrysophyta (orange). Station m3s4 was characterized by a substantial presence of Dinophyceae (yellow) and Cryptophyceae (orange), indicating a diverse phytoplankton community. Station m4s4 shows a similar trend with a dominance of Chrysophyta and a notable increase in Dinophyta.

In the CHEMTAX analysis of the algal group composition for matching LM23, stations showed that the Diatoms were consistently dominant across all stations except m3s4, corroborating the findings from the microscopy analysis. The CHEMTAX results shown a higher contribution of Chlorophyceae (green) and Cryptophytes (brown) across several stations, particularly at station m1s5 and m3s4. Cyanobacteria (blue) shown significant presence at stations m1s3 and m3s4, similar to the microscopy results. Dinophyceae (yellow) and Crypsophytes (orange) were also prominent, particularly in the later stations (m3s4 and m4s4), aligning with the microscopy determinations observations.

**Figure 9:**
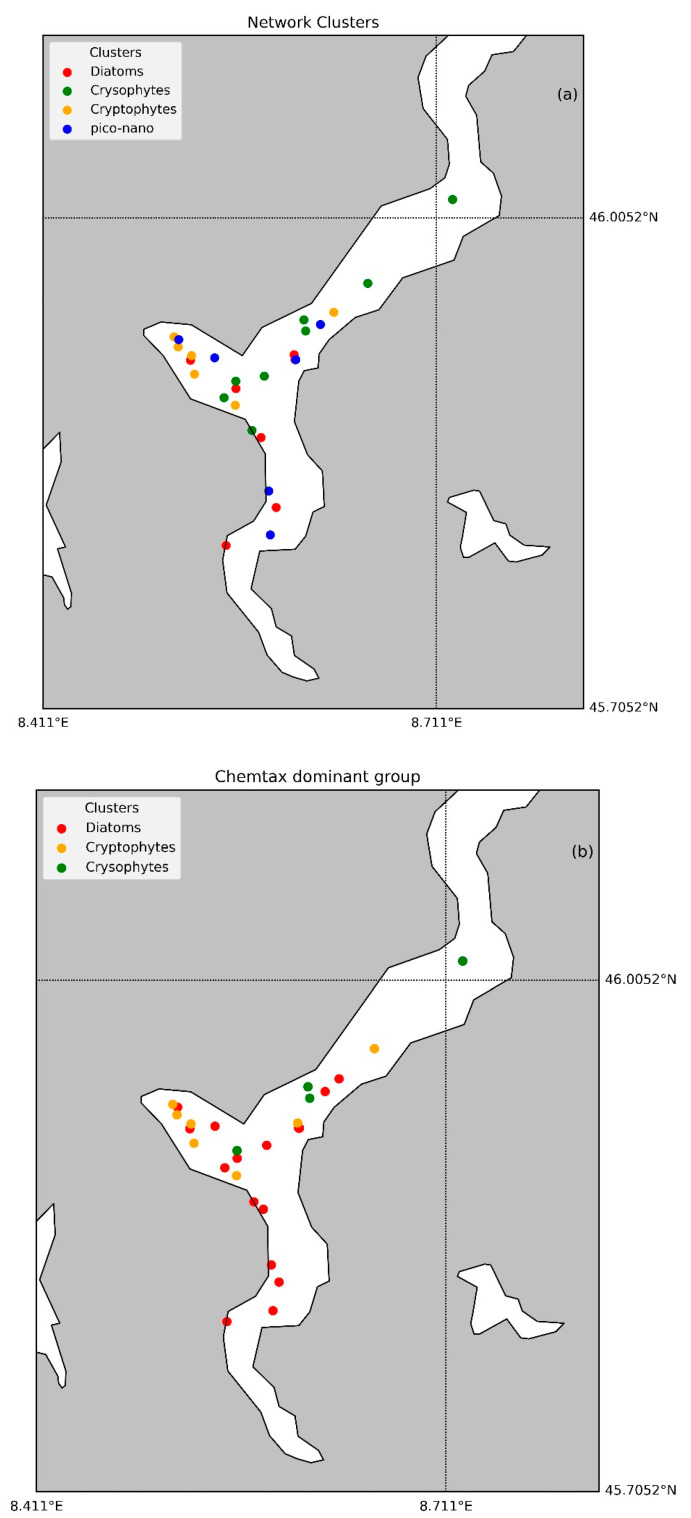
Dominant phytoplankton groups at each station as identified by Network (**a**) and CHEMTAX (**b**) analysis. Stations are color-coded to indicate the prevailing algal groups: red for diatoms, green for Chrysophyceae, yellow for Cryptophyceae, and blue for pico-nano mixed fraction (only in Network analysis).

The network community analysis of phytoplankton pigments yielded a modularity score of 0.18, indicating a relatively weak community structure. Modularity measures the strength of division within a network into distinct communities, with higher values reflecting a clearer separation. Four primary pigment communities were identified through this analysis. However, Fuco emerged as the dominant pigment at nearly all sampling stations. To refine the community identification, the results were re-evaluated using CHEMTAX. According to CHEMTAX, the refined community structure includes Diatoms, Chrysophyceae, Cryptophyceae, and a fourth community not clearly distinguished. Specifically, Diatoms were identified as the primary community due to the prevalence of Fuco, while chrysophytes were recognized for their characteristic pigments. Cryptophyceae, on the other hand, did not show a clear single pigment correlation, leading to the absence of a distinct classification for this group. The spatial distribution of dominant phytoplankton groups at different stations in Lake Maggiore can be analyzed by using two distinct analytical methods: CHEMTAX analysis and network analysis (Figure 9). Accordingly, in the CHEMTAX classification, Diatoms dominate most stations, particularly in the southern and central parts of the lake, which corresponds with their typical prevalence in nutrient-rich environments. In contrast, Cryptophyceae are more dominant in certain northern stations, suggesting variations in local environmental conditions that favor different phytoplankton communities. The results of network analysis, clusters stations based on similarities in phytoplankton community structure, were categorized with the same color scheme as the CHEMTAX map but include an additional group, pico-nano (blue). The network clusters provide a more nuanced perspective, grouping stations together based on the overall similarity of their phytoplankton communities rather than the dominance of a single group. Comparing the two maps, it is evident that Diatoms were a predominant group across many stations in Lake Maggiore. However, the maps also highlight the spatial heterogeneity of the phytoplankton community.

## 4. Discussion

The analysis of LM23 pigments dataset of Lake Maggiore provided insights into the dynamic and diverse nature of the lake’s phytoplankton community, reflecting its oligotrophic state and seasonal variations. The observed variability in key pigments, such as TChl *a* and Fuco, points to significant spatial and temporal shifts in primary productivity and community composition. The current study’s findings align with those from the 2022 CIPAIS report and the previous literature, confirming that diatoms dominate the phytoplankton community during different times of the year [12,68]. This seasonal succession is typical of oligotrophic lakes, where Diatoms thrive during cooler nutrient-rich conditions, while Cyanobacteria and Dinophyceae take over in warmer stratified waters [13,67]. The ANOVA analysis highlights the seasonal effect, with TChl *a* concentrations being significantly higher in spring compared to fall. This finding aligns with our observations of diatom dominance during the spring months, emphasizing how nutrient availability during this period fosters robust primary productivity. Additionally, the significant variations in Echin across seasons further supported the seasonal shifts in community composition, highlighting how environmental conditions affect phytoplankton dynamics. The detection of Zea, a cyanobacteria marker, in 68% of the samples suggests a consistent presence of small cyanobacteria species throughout the year, while analysis based only on in our superficial samples did not capture the cyanobacterial dominance that can occur during late summer. The presence of *Anabaena* species, as indicated by Echin, further reinforces the notion of a diverse cyanobacterial community. However, the absence of pigments such as Myxo and Gyro, typically associated with *Microcystis* species, despite microscopy findings, suggests that these cyanobacteria may be present in low abundance [4].

The spatial analysis showed that diatom-related pigments like Fuco exhibited significant variability, likely due to localized environmental conditions such as nutrient availability and water column stability [11]. The patchy distribution of Peri, a dinoflagellate marker, suggests episodic blooms driven by specific triggers, such as increased stratification and light availability in summer [5]. The PCA results provided additional context, revealing shifts in phytoplankton community health, particularly the inverse relationship between Chlide *a*, a marker of senescence, and Fuco, indicating changes in diatom vitality throughout the sampling period. The absence of significant markers for prasinophytes and haptophytes, such as But and Hex, suggests that these groups play a lesser role in Lake Maggiore compared to other taxa, reinforcing the findings of earlier studies [9,13]. In terms of phytoplankton size fractions, the dominance of micro-phytoplankton in high-TChl *a* samples highlights the importance of larger phytoplankton in driving productivity, especially during periods of increased nutrient availability. Meanwhile, nano- and pico-phytoplankton maintained a stable presence, underscoring their adaptability to nutrient-poor conditions and their contribution to the lake’s overall biodiversity [12]. The CHEMTAX analysis largely confirmed the results from PCA and HCA, with diatoms emerging as the dominant group. The diatom bloom observed in early spring, followed by an increase in cryptophytes and large dinoflagellates by mid-summer, mirrors seasonal trends noted in the Ghiffa station data [11].

Buchaca et al. [25] suggested that in oligotrophic systems like Lake Maggiore, caution was needed when using CHEMTAX, particularly under low-irradiance or nutrient-replete conditions, such as those found beneath ice cover or, in our case, during the stratified summer months. CHEMTAX may overestimate the biovolume of certain taxa, particularly chlorophytes, when their abundance is low and colony-forming species are present. This insight mirrors the discrepancies observed in our study, where CHEMTAX and microscopy findings for chlorophytes diverged in late summer-autumn samples. CHEMTAX’s pigment-based classification offers a broader perspective on community composition, while microscopy provides more detailed taxonomic insights, a combination that provided better understanding in spatio-temporal phytoplankton dynamics in complex ecosystems like Lake Maggiore [68,69]. While the CHEMTAX map gives a direct view of the dominant phytoplankton group at each station, the network analysis adds depth by showing how stations with similar community structures were related, revealing potential underlying ecological or environmental gradients. This highlights the dynamic nature of the lake’s ecosystem and the influence of local conditions on phytoplankton communities. This analysis, paired with previous studies, suggests that the lake’s phytoplankton composition was heavily influenced by environmental drivers such as nutrient fluxes, water temperature, and light availability, with spatial and temporal variations adding further complexity [9].

To improve monitoring and understanding of phytoplankton dynamics in Lake Maggiore, a combination of in situ and satellite-based approaches should be considered. In situ methods, such as pigment analysis and the application of CHEMTAX algorithm, offer detailed insights into the composition of phytoplankton communities, but they can be resource-intensive and provide only limited spatial and temporal coverage. Satellite remote sensing, on the other hand, can offer continuous large-scale observations, particularly of surface chlorophyll concentrations, which can serve as a proxy for phytoplankton biomass. Recent advances in hyperspectral sensors allow for the detection of specific phytoplankton groups, including diatoms and cyanobacteria, making remote sensing an increasingly valuable tool for ecosystem monitoring. Combining these approaches would provide a more comprehensive assessment of phytoplankton variability and productivity [38,39]. In situ data could be used to calibrate and validate satellite-derived estimates, ensuring greater accuracy in detecting phytoplankton blooms and understanding their drivers. Additionally, integrating physical and chemical data, such as water temperature, nutrient levels, and stratification patterns, would help to link phytoplankton dynamics with environmental conditions, offering insights into the ecological mechanisms that determine community composition.

## 5. Conclusions

This study applied chemotaxonomy through HPLC pigment analysis and the CHEMTAX algorithm to provide a detailed characterization of the phytoplankton community in Lake Maggiore. The findings revealed a dynamic seasonal succession dominated by diatoms in cooler nutrient-rich periods and cyanobacteria and dinoflagellates during warmer stratified conditions. Despite the presence of cyanobacteria throughout the year, 2024 did not exhibit the late-summer dominance seen in previous years, suggesting interannual variability influenced by environmental factors.

The integration of PCA, HCA, and microscopy with pigment data allowed for a comprehensive assessment of phytoplankton diversity and confirmed the suitability of chemotaxonomy as a complementary method to traditional microscopic techniques. However, discrepancies in chlorophyte abundance between CHEMTAX and microscopy highlight the need for cautious interpretation, particularly under conditions of low abundance. The study also underscores the importance of continuous monitoring, especially for detecting subtle shifts in community composition due to climate-driven changes in lake conditions.

Moving forward, the combination of in situ methods with satellite remote sensing holds promise for more comprehensive and efficient monitoring of phytoplankton dynamics and HABs in Lake Maggiore [70]. Future efforts should focus on calibrating satellite-derived estimates with in situ data to enhance the accuracy of bio-optical algorithms for detecting and monitoring phytoplankton blooms and their ecological drivers. These insights are crucial for developing strategies to mitigate the impacts of HABs and maintain the ecological integrity of Lake Maggiore.

## Figures and Tables

**Table 1:** Pigment concentrations (ng/inj) and ratios of *Anabaena* sp. PCC 7120 and *Microcystis aeruginosa*.

	*Microcystis aeruginosa*			*Anabaena PCC 7120*		
	Amount [ng/inj]	Ratio%: TChl *a*	Ratio%: β Caro	Amount [ng/inj]	Ratio%: TChl *a*	Ratio%: β-caro
Myxo	6.3	3.0	37.3	2.4	1.3	8.3
Zea	27.1	12.9	160.4	2.1	1.1	7.2
Gyro	2.1	1.0	12.4	3.1	1.6	10.7
Echin	5.2	2.5	30.8	22.4	11.7	77.2
β-caro	16.9	8.1	100	29	15.2	100.0
TChl *a*	209.2	100		190.8	100	

**Table 2:** Selected statistical data characterizing the variability of the LM23 dataset: the average (mean) value along with the variation coefficient. The number of measurements falling below LOD for the pigments was also reported.

Parameters	Mean	CV%	Max	Min	Below Detection Limit
TChl *a* [mg/m^3^]	2.933	54.7	6.901	1.133	-
TChl *b* [mg/m^3^]	0.078	42.	0.155	0.024	-
TChl *c* [mg/m^3^]	0.197	55.2	0.442	0.077	-
Fuco [mg/m^3^]	0.513	55.9	1.101	0.145	-
Zea [mg/m^3^]	0.199	42.6	0.404	0.097	-
Peri [mg/m^3^]	0.136	95.2	0.491	0	1
Allo [mg/m^3^]	0.146	52.3	0.336	0.023	-
Diato [mg/m^3^]	0.040	58.1	0.08	0	4
Caro [mg/m^3^]	0.195	55.9	0.509	0.076	-
Echin [mg/m^3^]	0.038	54.2	0.071	0	2
SPM [g/m^3^]	0.011	38.9	0.025	0.007	
T [°C]	20.53	17.02	25.62	15.5	
Depth [m]	222.6	49.7	383	60	
LAT			45.559	45.48186	
LON			8.347	8.30507	

**Table 3:** ANOVA analysis and Tukey test for the analysis of seasonal variation in Lake Maggiore.

Variable	ANOVA F-Value	ANOVA *p*-Value	Tukey Significant Differences (*p* < 0.05)
TChl *a*	6.31	0.0063	Fall vs. Spring (*p* = 0.0045)
TChl *b*	0.9	0.4204	None
TChl *c*	11.38	0.0003	Fall vs. Spring (*p* = 0.0003), Fall vs. Summer (*p* = 0.0492), Spring vs. Summer (*p* = 0.0297)
Fuco	3.54	0.045	Fall vs. Spring (*p* = 0.0357)
Zea	8.39	0.0017	Fall vs. Spring (*p* = 0.0015)
Peri	13.28	0.0001	Fall vs. Spring (*p* = 0.0001), Fall vs. Summer (*p* = 0.0216), Spring vs. Summer (*p* = 0.0267)
Allo	7.92	0.0023	Fall vs. Spring (*p* = 0.0165), Spring vs. Summer (*p* = 0.0041)
Diato	1.19	0.3221	None
Caro	8.44	0.0017	Fall vs. Spring (*p* = 0.0014)
Echin	4.18	0.0276	Fall vs. Spring (*p* = 0.0250)
SPM	2.16	0.1369	None

**Table 4:** F1 Refined CHEMTAX matrix (from initial F0, Schlüter et al. [20]).

	Chl *c*1	Peri	Fuco	Neo	Viola	Allo	Lut	Zea	Echin	TChl_*b*
Diatoms	0.021	0.000	0.039	0.000	0.000	0.000	0.000	0.005	0.000	0.000
Chlorophyceae	0.000	0.000	0.000	0.030	0.025	0.000	0.099	0.001	0.000	0.172
Cyanophytes	0.000	0.000	0.000	0.000	0.000	0.000	0.000	0.284	0.055	0.000
Crysophytes	0.000	0.000	0.207	0.000	0.094	0.000	0.000	0.001	0.000	0.000
Dinophyceae	0.000	0.334	0.000	0.000	0.000	0.000	0.000	0.000	0.000	0.000
Cryptophytes	0.000	0.000	0.000	0.000	0.000	0.143	0.000	0.000	0.000	0.000

## Data Availability

The data will be made available on a reasonable request.

## Supplementary Materials

The following supporting information can be downloaded at: https://www.mdpi.com/article/10.3390/microorganisms12112211/s1, Figure S1: TChl a concentrations (mg/m^3^) and the correspondent bottom-depth for the LM23 stations; Figure S2: Temporal variations in TChl a concentrations (mg/m^3^) across different months with reference (dashed grey vertical line) to the station collected close to the 10:00 UTC time; Figure S3: Principal Component analysis of pigments and main environmental variables; Table S1: CHEMTAX F0 matrix from Schlüter et al. [20].

## References

[1] Mishra S., Stumpf R., Schaeffer B., Werdell P., Loftin K., Andrew M. (2019). Measurement of Cyanobacterial Bloom Magnitude using Satellite Remote Sensing. Sci. Rep..

[2] Havens K.E., Hudnell H.K. (2008). Cyanobacteria blooms: Effects on aquatic ecosystems. Cyanobacterial Harmful Algal Blooms: State of the Science and Research Needs.

[3] Dörnhöfer K., Klinger P., Heege T., Oppelt N. (2018). Multi-sensor satellite and in situ monitoring of phytoplankton development in a eutrophic-mesotrophic lake. Sci. Total Environ..

[4] Bresciani M., Giardino C., Lauceri R., Matta E., Cazzaniga I., Pinardi M., Lami A., Austoni M., Viaggiu E., Congestri R. (2016). Earth observation for monitoring and mapping of cyanobacteria blooms. Case studies on five Italian lakes. J. Limnol..

[5] Mosello R., Ambrosetti W., Arisci S., Bettinetti R., Buzzi F., Calderoni A., Carrara E., de Bernardi R., Galassi S., Garibaldi L. (2010). Trend of water quality of the deep subalpine lakes in relation to anthropic pressure and climate - Evoluzione recente della qualità delle acque dei laghi profondi sudalpini (Maggiore, Lugano, Como, Iseo e Garda) in risposta alle pressioni antropiche e alle variazioni climatiche. Biol. Ambient..

[6] Salmaso N., Capelli C., Shams S., Cerasino L. (2015). Expansion of bloom-forming *Dolichospermum lemmermannii* (Nostocales, Cyanobacteria) to the deep lakes south of the Alps: Colonization patterns, driving forces and implications for water use. Harmful Algae.

[7] Salmaso N., Boscaini A., Pindo M. (2020). Unraveling the Diversity of Eukaryotic Microplankton in a Large and Deep Perialpine Lake Using a High Throughput Sequencing Approach. Front. Microbiol..

[8] Mosello R., Ruggiu D. (1985). Nutrient load, trophic conditions and restoration prospects of Lake Maggiore. Int. Rev. Ges. Hydrobiol. Hydrogr..

[9] Ruggiu D., Morabito G., Panzani P., Pugnetti A. (1998). Trends and relations among basic phytoplankton characteristics in the course of the long-term oligotrophication of Lake Maggiore (Italy). Hydrobiologia.

[10] Marziali L., Guzzella L., Salerno F., Marchetto M., Valsecchi L., Tasselli S., Roscioli C., Schiavon A. (2021). Twenty-year sediment contamination trends in some tributaries of Lake Maggiore (Northern Italy): Relation with anthropogenic factors. Environ. Sci. Pollut. Res..

[11] CIPAIS (Commissione Internazionale per la Protezione delle Acque Italo-Svizzere), CNR-IRSA, Commissione Internazionale per la Protezione Delle Acque Italo-Svizzere (2023). Ricerche Sull’evoluzione del Lago Maggiore.

[12] Kamenir Y., Morabito G. (2009). Lago Maggiore oligotrophication as seen from the long-term evolution of its phytoplankton taxonomic size structure. J. Limnol..

[13] Morabito G., Oggioni A., Austoni M., Salmaso N., Naselli-Flores L., Cerasino L., Flaim G., Tolotti M., Padisák J. (2012). Resource ratio and human impact: How diatom assemblages in Lake Maggiore responded to oligotrophication and climatic variability. Phytoplankton Responses to Human Impacts at Different Scales.

[14] Austoni M., Marchetto A., Commissione Internazionale per la Protezione Delle Acque Italo-Svizzere (2019). Struttura delle Associazioni Fitoplanctoniche nel Lago Maggiore e loro Modificazioni in Relazione a Fattori di Controllo Trofici e Climatici in CNR IRSA.

[15] HELCOME (2023). Guidelines for Monitoring of Phytoplankton Species Composition, Abundance and Biomass.

[16] Vidussi F., Claustre H., Manca B.B., Luchetta A., Marty J.C. (2001). Phytoplankton pigment distribution in relation to upper thermocline circulation in the eastern Mediterranean Sea during winter. J. Geophys. Res..

[17] Uitz J., Claustre H., Morel A., Hooker S.B. (2006). Vertical distribution of phytoplankton communities in open ocean: An assessment based on surface chlorophyll. J. Geophys. Res..

[18] Hirata T., Hardman-Mountford N.J., Brewin R.J.W., Aiken J., Barlow R.G., Suzuki K., Isada T., Howell E., Hashioka T., Noguci-Aita M. (2011). Synoptic relationships between surface chlorophyll-a and diagnostic pigments specific to phytoplankton functional types. Biogeosciences.

[19] Schlüter L., Garde K., Kaas H. (2004). Detection of the toxic cyanobacteria *Nodularia spumigena* by means of a 4-keto-myxoxanthophyll-like pigment in the Baltic Sea. Mar. Ecol. Prog. Ser..

[20] Schlüter L., Behl B., Striebel M., Stibor H. (2016). Comparing microscopic counts and pigment analyses in 46 phytoplankton communities from lakes of different trophic state. Freshw. Biol..

[21] Lauceri R., Bresciani M., Lami A., Morabito G. (2018). Chlorophyll a interference in phycocyanin and allophycocyanin spectrophotometric quantication. J. Limnol..

[22] Wojtasiewicz B., Stoń-Egiert J. (2016). Bio-optical characterization of selected cyanobacteria strains present in marine and freshwater ecosystems. J. Appl. Phycol..

[23] Roy S., Llewellyn C.A., Egeland E.S., Johnsen G. (2011). Phytoplankton Pigments, Characterization, Chemotaxonomy and Applications in Oceanography.

[24] Mackey M., Mackey D., Higgins H.W., Wright S.W. (1996). CHEMTAX—A program for estimating class abundances from chemical markers: Application to HPLC measurements of phytoplankton. Mar. Ecol. Prog. Ser..

[25] Buchaca T., Felip M., Catalan J. (2005). A comparison of HPLC pigment analyses and biovolume estimates of phytoplankton groups in an oligotrophic lake. J. Plankton Res..

[26] Guisande C., Barreiro A., Acuna A., Marciales L.J., Hernandez E., Torres A.M., Aranguren N., López W., Duque S.R., Gallo L.J. (2008). Testing of the CHEMTAX program in contrasting Neotropical lakes, lagoons, and swamps. Limnol. Oceanogr.-Methods.

[27] Lauridsen T.L., Schlüter L., Johansson L.S. (2011). Determining algal assemblages in oligotrophic lakes and streams: Comparing information from newly developed pigment/chlorophyll a ratios with direct microscopy. Freshw. Biol..

[28] Goela P.C., Danchenko S., Icely J.D., Lubian L.M., Cristina S., Newton A. (2014). Using CHEMTAX to evaluate seasonal and interannual dynamics of the phytoplankton community off the south-west coast of Portugal. Estuarine. Coast. Shelf Sci..

[29] Agirbas E., Feyzioglu A.M., Kopuz U., Llewellyn C.A. (2015). Phytoplankton community composition in the south-eastern Black Sea determined with pigments measured by HPLC-CHEMTAX analyses and microscopy cell counts. J. Mar. Biol. Assoc. UK.

[30] Lee M., Won N.-I., Baek S.H. (2020). Comparison of HPLC Pigment Analysis and Microscopy in Phytoplankton Assessment in the Seomjin River Estuary, Korea. Sustainability.

[31] Saggiomo M., Bolinesi F., Brunet C., Passarelli A., Margiotta F., Saggiomo V., Mangoni O. (2023). A CHEMTAX-derived phytoplankton community structure during 12-year observations in the Gulf of Naples (LTER-MC). Mar. Ecol..

[32] Irigoien X., Meyer B., Harris R., Harbour D. (2004). Using HPLC pigment analysis to investigate phytoplankton taxonomy: The importance of knowing your species. Helgol. Mar. Res..

[33] Tamm M., Freiberg R., Tõnno I., Nõges P., Nõges T. (2015). Pigment-based chemotaxonomy—A quick alternative to determine algal assemblages in large shallow eutrophic lake?. PLoS ONE.

[34] Simmons L., Sandgren C., Berges J. (2016). Problems and pitfalls in using HPLC pigment analysis to distinguish Lake Michigan phytoplankton taxa. J. Great Lakes Res..

[35] Hernández-Avilés J.S., Callieri C., Bertoni R., Morabito G., Leoni B., Lepori F., Buzzi F., Salmaso N. (2018). Prokaryoplankton and phytoplankton community compositions in five large deep perialpine lakes. Hydrobiologia.

[36] Morabito G., Austoni M., Commissione Internazionale per la Protezione Delle Acque Italo-Svizzere (2016). Caratteristiche Strutturali delle Associazioni Fitoplanctoniche nel Lago Maggiore ed Evoluzione Stagionale dei Popolamenti-C.N.R.-I.S.E..

[37] Rogora M., Austoni M., Caroni R., Giacomotti P., Kamburska L., Marchetto A., Mosello R., Orru’ A., Tartari G., Dresti C. (2021). Temporal changes in nutrients in a deep oligomictic lake: The role of external loads versus internal processes. J. Limnol..

[38] Bernard S., Kudela R., Robertson Lain L., Pitcher G.C., IOCCG (2021). Observation of Harmful Algal Blooms with Ocean Colour Radiometry.

[39] Kutser T., Metsamaa L., Strömbeck N., Vahtmäe E. (2006). Monitoring cyanobacterial blooms by satellite remote sensing. Estuar. Coast. Shelf Sci..

[40] Simis S.G.H., Peters S., Gons H.J. (2005). Remote sensing of the cyanobacterial pigment phycocyanin in turbid inland water. Limnol. Oceanogr..

[41] Ambrosetti W., Barbanti L. (1999). Deep water warming in lakes: An indicator of climatic change. J. Limnol..

[42] Ambrosetti W., Barbanti L., Rolla A., Castellano L., Sala N. (2012). Hydraulic paths and estimation of the real residence time of the water in Lago Maggiore (N Italy): Application of massless markerstransported in 3D motion fields. J. Limnol..

[43] Fenocchi A., Rogora M., Sibilla S., Ciampittiello M., Dresti C. (2018). Forecasting the evolution in the mixing regime of a deep subalpine lake under climate change scenarios through numerical modelling (Lake Maggiore, Northern Italy/Southern Switzerland). Clim. Dyn..

[44] Rogora M., Buzzi F., Dresti C., Leoni B., Lepori F., Mosello R., Patelli M., Salmaso N. (2018). Climatic effects on vertical mixing and deep-water oxygen content in the subalpine lakes in Italy. Hydrobiologia.

[45] Van Heukelem L., Thomas C.S. (2001). Computer-assisted high-performance liquid chromatography method development with applications to the isolation and analysis of phytoplankton pigments. J. Chromatogr. A.

[46] Canuti E. (2023). Phytoplankton pigment in situ measurements uncertainty evaluation: An HPLC interlaboratory comparison with a European-scale dataset. Front. Mar. Sci..

[47] Hooker S.B., Van Heukelem L., Thomas C.S., Claustre H., Ras J., Barlow R., Sessions H., Schluter L., Perl J., Trees C. (2005). The Second SeaWIFS HPLC Analysis Round-Robin Experiment/SeaHARRE-2; NASA/TM-2005-212785. https://oceancolor.gsfc.nasa.gov/fsg/hplc/SH2_TM2005_212785.pdf.

[48] Brewin R.J.W., Sathyendranath S., Hirata T., Lavender S.J., Barciela R.M., Hardman-Mountford N.J. (2010). A three-component model of phytoplankton size class for the Atlantic Ocean. Ecol. Model..

[49] Punginelli C., Wilson A., Routaboul J.M., Kirilovsky D. (2009). Influence of zeaxanthin and echinenone binding on the activity of the Orange Carotenoid Protein. Biochim. Biophys. Acta (BBA)-Bioenerg..

[50] Takaichi S., Mochimaru M. (2007). Carotenoids and carotenogenesis in cyanobacteria: Unique ketocarotenoids and carotenoid glycosides. Cell. Mol. Life Sci..

[51] Callieri C., Bertoni R., Contesini M., Bertoni F. (2014). Lake level fluctuations boost toxic cyanobacterial “oligotrophic blooms”. PLoS ONE.

[52] Aiken J., Pradhan Y., Barlow R., Lavender S., Poulton A., Holligan P., Hardman-Mountford N. (2009). Phytoplankton pigments and functional types in the Atlantic Ocean: A decadal assessment, 1995–2005. Deep. Sea Res. Part II Top. Stud. Oceanogr..

[53] Trees C.C., Clark D.K., Bidigare R.R., Ondrusek M.E., Mueller J.L. (2000). Accessory pigments versus chlorophyll a concentrations within the euphotic zone: A ubiquitous relationship. Limnol. Oceanogr..

[54] Latasa M., Bidigare R.R. (1998). A comparison of phytoplankton populations of the Arabian Sea during the Spring Intermonsoon and Southwest Monsoon of 1995 as described by HPLC-analyzed pigments. Deep. Sea Res. Part II Top. Stud. Oceanogr..

[55] Catlett D., Siegel D.A. (2018). Phytoplankton pigment communities can be modeled using unique relationships with spectral absorption signatures in a dynamic coastal environment. J. Geophys. Res. Ocean..

[56] Anderson C.R., Siegel D.A., Brzeniski M.A., Guillocheau N. (2008). Controls on temporal patterns in phytoplankton community structure in the Santa Barbara Channel, California. J. Geophys. Res..

[57] Kramer S.J., Siegel D.A. (2019). How can phytoplankton pigments be best used to characterize surface ocean phytoplankton groups for ocean color remote sensing algorithms?. J. Geophys. Res. Ocean..

[58] Kramer S.J., Siegel D.A., Graff J.R. (2020). Phytoplankton Community Composition Determined From Co-variability Among Phytoplankton Pigments From the NAAMES Field Campaign. Front. Mar. Sci..

[59] Canuti E., Penna A. (2024). Dynamics of Phytoplankton Communities in the Baltic Sea: Insights from a Multi-dimensional Analysis of Pigment and Spectral Data: Part I, Pigment Dataset. Front. Mar. Sci. Sec. Ocean. Obs..

[60] Zhang B., Horvath S. (2005). A general framework for weighted gene co-expression network analysis. Stat. Appl. Genet. Mol. Biol..

[61] Blondel V., Guillaume J.-L., Lambiotte R., Lefebvre E. Fast unfolding of communities in large networks. J. Stat. Mech. Theory Exp..

[62] Utermöhl H. (1958). Zur vervollkommnung der quantitativen phytoplankton-methodik. Mitt. Int. Verein. Limnol..

[63] Lund J.W.G., Kipling C., Le Cren E.D. (1958). The inverted microscope method of estimating algal number and the statistical basis of estimations by counting. Hydrobiologia.

[64] (2006). Water Quality-Guidance Standard on the Enumeration of Phytoplankton Using Inverted Microscopy (Utermöhl Technique).

[65] Hillebrand H., Dürselen C.C.-D., Kirschtel D., Pollingher U., Zohary T. (1999). Biovolume calculation for pelagic and benthic microalgae. J. Phycol..

[66] Sun J., Liu D. (2003). Geometric models for calculating cell biovolume and surface area for phytoplankton. J. Plankton Res..

[67] (2015). Water Quality-Guidance on the Estimation of Phytoplankton Biovolume.

[68] Salmaso N. (2000). Factors affecting the seasonality and distribution of cyanobacteria and chlorophytes: A case study from the large lakes south of the Alps, with special reference to Lake Garda. Hydrobiologia.

[69] Austoni M., Giacomotti P., Kamburska L., Lami A., Manca D., Marchetto A., Commissione Internazionale per la Protezione Delle Acque Italo-Svizzere (2022). Struttura delle Associazioni Fitoplanctoniche nel Lago Maggiore e loro Modificazioni in Relazione a Fattori di Controllo Trofici e Climatici in CNR IRSA.

[70] Giardino C., Bresciani M., Stroppiana D., Oggioni A., Morabito G. (2014). Optical remote sensing of lakes: An overview on Lake Maggiore. J. Limnol..

