# Supplementary material for: Characterization of Phytoplankton Composition in Lake Maggiore: Integrated Chemotaxonomy for Enhanced Cyanobacteria Detection
Source: Microorganisms. 2024 Oct 31;12(11):2211. doi: 10.3390/microorganisms12112211 (PMC11596642; doi:10.3390/microorganisms12112211)

Supplementary Table S1. CHEMTAX F0 matrix from Schlüter et al. (2016).

Pigment Selection	1	1	1	1	1	1	1	1	1	1	1
Class / Pigment	Chl_c1	Peri	Fuco	Neo	Viola	Allo	Lut	Zea	Echin	Chl_b	Chl_a
Diatoms	0.018	0	0.036	0	0	0	0	0.005	0	0	1
Chlorophyceae	0	0	0	0.038	0.036	0	0.119	0.001	0	0.2771	1
Cyaonophytes	0	0	0	0	0	0	0	0.427	0.071	0	1
Crysophytes	0	0	0.283	0	0.134	0	0	0.001	0	0	1
Dinophyceae	0	0.501	0	0	0	0	0	0	0	0	1
Cryptophytes	0	0	0	0	0	0.156	0	0	0	0	1

Supplementary Figure S1. TChl *a* concentrations (mg/m³) and the correspondent bottom-depth for the LM23 stations.

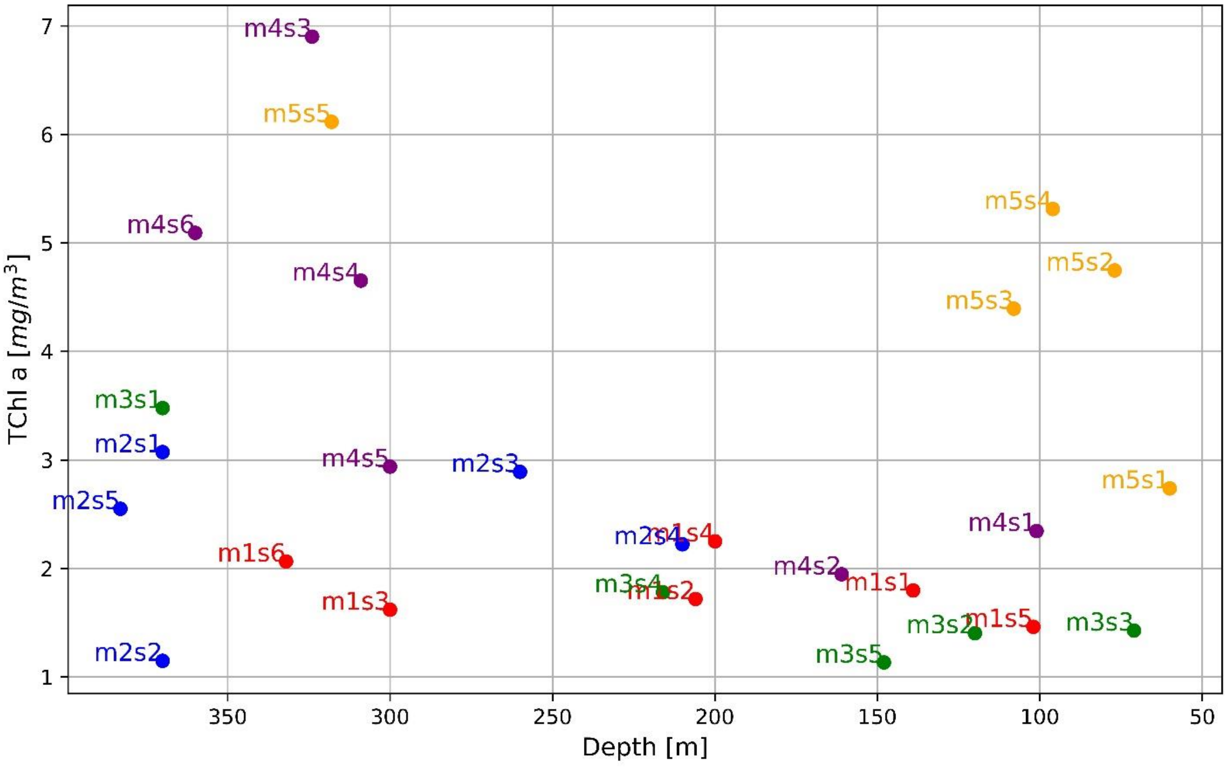

Supplementary Figure S2. Temporal variations in TChl *a* concentrations (mg/m³) across different months with reference (dashed grey vertical line) to the station collected close to the 10:00 UTC time

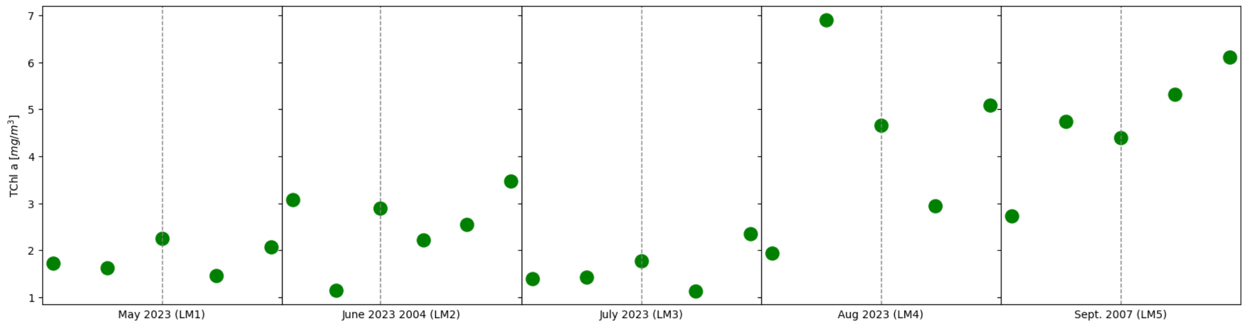

Supplementary Figure S3. Principal Component analysis of pigments and main environmental variables

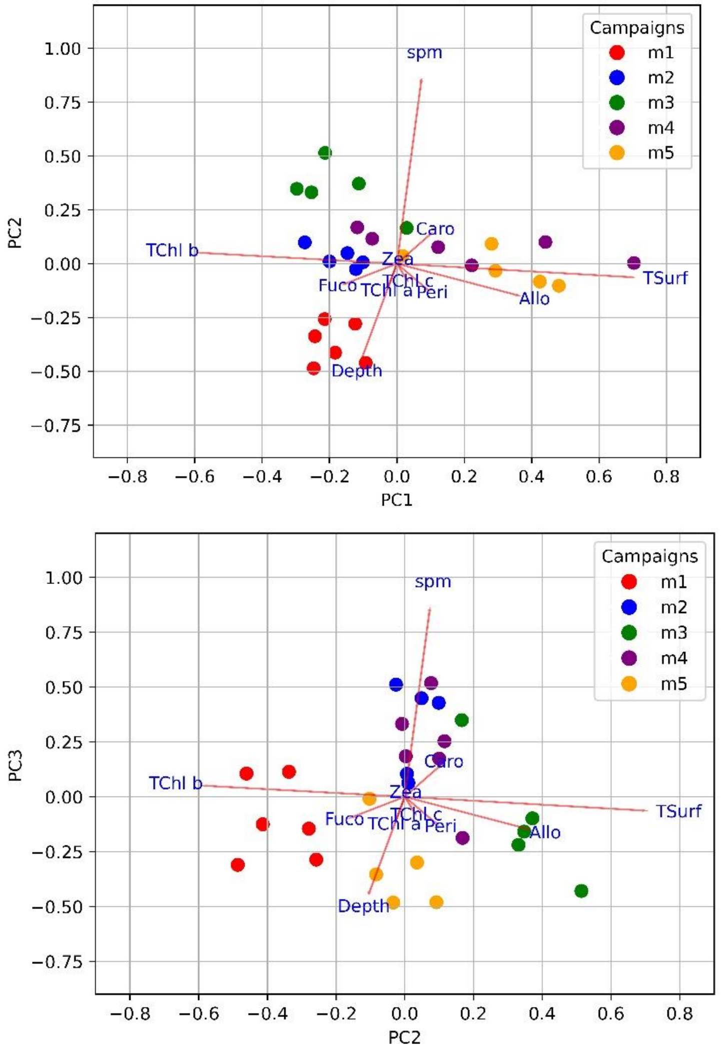

Supplement: Supplementary file 1 [file microorganisms-12-02211-s001.zip › microorganisms-3267889-supplementary.pdf]
